# Assessing Illumina technology for the high-throughput sequencing of bacteriophage genomes

**DOI:** 10.7717/peerj.2055

**Published:** 2016-06-01

**Authors:** Branko Rihtman, Sean Meaden, Martha R.J. Clokie, Britt Koskella, Andrew D. Millard

**Affiliations:** 1 School of Life Sciences, University of Warwick, Coventry, United Kingdom; 2 College of Life and Environmental Sciences, University of Exeter, United Kingdom; 3 Department of Infection, Immunity and Inflammation, University of Leicester; 4 Department of Integrative Biology, University of California, Berkeley, California, United States; 5 Warwick Medical School, University of Warwick, United Kingdom

**Keywords:** Bacteriophage, Genome, Assembly, Sequencing, Illumina

## Abstract

Bacteriophages are the most abundant biological entities on the planet, playing crucial roles in the shaping of bacterial populations. Phages have smaller genomes than their bacterial hosts, yet there are currently fewer fully sequenced phage than bacterial genomes. We assessed the suitability of Illumina technology for high-throughput sequencing and subsequent assembly of phage genomes. In silico datasets reveal that 30× coverage is sufficient to correctly assemble the complete genome of ~98.5% of known phages, with experimental data confirming that the majority of phage genomes can be assembled at 30× coverage. Furthermore, in silico data demonstrate it is possible to co-sequence multiple phages from different hosts, without introducing assembly errors.

## Introduction

Viruses are the most abundant biological entities on the planet, having a ubiquitous distribution in all known biological niches. They play a crucial role in every environment they occupy, influencing the growth and metabolism of the hosts they infect (Ankrah et al., 2014; Mann et al., 2003; Meaden, Paszkiewicz & Koskella, 2015; Thompson et al., 2011), changing the chemical balance of the surrounding environment (Weitz & Wilhelm, 2012), affecting the diversity of the incumbent microbial community (Weitz et al., 2015), and driving microbial evolution through the transfer of genetic material (Lindell et al., 2005; Millard et al., 2004). In the marine environment, viruses are found at up to ~1 × 10^8^ ml^−1^ of seawater, with an estimated ~4 × 10^30^ viruses in the oceans (Suttle, 2005). Phages play an important role in biogeochemical cycling, diverting the flow of carbon to dissolved organic matter and particulate organic matter through the lysis of their bacterial hosts, thus influencing the amount of carbon that is sequestered to the deep ocean by the biological pump (Suttle, 2007). In soils, viral abundance is estimated to be 10^8^–10^9^ g^−1^ (Williamson et al., 2013). The human gut is estimated to contain 10^15^ phages, although the exact role of phages in shaping the incumbent bacterial community structure and human health is unknown (Dalmasso, Hill & Ross, 2014). Unlike many other environments, such as the open ocean (Hurwitz & Sullivan, 2013), the majority of phages that have been found in gut metagenomes are temperate rather than obligately lytic (Howe et al., 2015). These phages possibly play an active role in human gut immunity and metabolism by forming part of the mucus-associated microbiome, where they may serve as a first line of defence against invading bacteria (Barr et al., 2013; Barr et al., 2015). In the mouse gut, phages have been implicated in providing a mechanism for multidrug resistance via reservoir genes (Modi et al., 2013). However, phages also hold promise as a possible alternative to antibiotics which are becoming ineffective with the increasing rise of antimicrobial resistance (Nobrega et al., 2015). In recent years, research of bacterial host immunity against phage infection has yielded one of the most promising genome-editing molecular techniques—the CRISPR-Cas system (Barrangou et al., 2007). Despite the importance of phages, there are relatively few isolates for which complete genome sequences are available. It is now over 30 years since the sequencing of the ssDNA phage ϕX174 in 1977 (Sanger et al., 1977), followed by the dsDNA phages lambda and T7 in the early 1980s (Dunn, Studier & Gottesman, 1983; Sanger et al., 1982).

The sequencing of these smaller phage genomes was completed many years before the first bacterial genome was published in 1995 (Fleischmann et al., 1995). In the last ten years, the number of bacterial and phage genomes has increased dramatically, coinciding with the decreasing cost-per-base of sequencing. Despite the smaller genome sizes of phages compared to their host, which should make them easier to sequence and assemble de novo, publicly available databases contain more finished bacterial than phage genomes. Whilst the numbers of finished bacterial genomes is slowly increasing (2,789 complete genomes within the European Nucleotide Archive—ENA, http://www.ebi.ac.uk/genomes/—at the time of writing), this is dwarfed by the tens of thousands of bacterial genomes that have been submitted as whole genome shotgun surveys (WGS) (e.g., ~40,000 *Salmonella* genomes alone). In comparison, there are 1,922 completely assembled phage genomes within the ENA, with no phage WGS assemblies in this database.

The vast majority of genes in viral metagenomes are not found in any of the currently sequenced phage genomes (Breitbart & Rohwer, 2005; Edwards & Rohwer, 2005; Hurwitz & Sullivan, 2013; Willner, Thurber & Rohwer, 2009). Increasing the number and diversity of sequenced phage isolates will improve our ability to assign unknown genes to a particular viral family. Combined with the additional information concerning the identity of the host they infect, the time of the year, and environmental conditions predominant at the location from which they were isolated, increasing the number of sequenced genomes will assist us in understanding the biology of a larger variety of novel phages. The recent sequencing of a small number of phage genomes has had a significant contribution towards understanding the bacterial metabolic processes in which they participate, highlighting the importance of further discovering the viral unknowns via sequencing of phage isolates (Chan et al., 2014; Chan et al., 2015; Kang et al., 2013; Roux et al., 2014; Sabehi et al., 2012; Zhao et al., 2013). The dearth of reference phage genomes infecting cells of a particular genera is highlighted by the estimation of 5,476 viral populations in the upper oceans, of which only 39 could be affiliated to cultured viruses (Brum et al., 2015). Re-sequencing of cultured phage genomes also provides insight into their genome evolution (Puxty et al., 2015). Altogether, it is clear that there exists a developing need for a high-throughput approach to the sequencing and re-sequencing of cultured phage genomes.

The limitations on the sequencing of phage isolates have previously been discussed in detail (Klumpp, Fouts & Sozhamannan, 2012). The largest single bottleneck will always be culturing of their host bacteria, which has been successfully circumvented by the use of metagenomics (Alavandi & Poornima, 2012; Blomstrom, 2011; Delwart, 2007; Edwards & Rohwer, 2005; Ge et al., 2013; Hurwitz & Sullivan, 2013; Kim, Whon & Bae, 2013). This development has greatly expanded our knowledge of phages, but there is ambiguity in identifying the hosts of phages found in metagenomes. Previously sequencing of phage genomes had been problematic due to cloning phage DNA for Sanger sequencing-based approaches, contaminating host DNA, and potential differences in %GC content relative to that of their hosts (Klumpp, Fouts & Sozhamannan, 2012), but these issues have been largely overcome by technological advances (Breitbart et al., 2003; Rohwer et al., 2001). Previous work, using 454 pyrosequencing and a column-based clean-up for DNA extraction has optimised methods for sequencing phages using this technology (Henn et al., 2010; Marine et al., 2011). Since then, 454 pyrosequencing technology has become largely obsolete with rapid advances in high-throughput sequencing technologies (see Loman et al. (2012), for a review). The Illumina MiSeq platform offers the potential to rapidly sequence hundreds of phage genomes. However, previous reports have suggested that Illumina data is of limited value for the de novo assembly of phage genomes (Klumpp, Fouts & Sozhamannan, 2012). Despite this, there are an increasing number of reports using Illumina technology for sequencing phage genomes (Carson et al., 2015; Malki et al., 2015; Smith et al., 2015; Tevdoradze et al., 2015). The resultant genomes have been assembled using a number of different assembly programs including SPAdes (Smith et al., 2015), Velvet (Cowley et al., 2015), and CLC Workbench (Carson et al., 2015) at a range of sequencing depths up to 18,000× coverage. Unlike their bacterial hosts, phages do not generally contain repetitive sequences such as gene duplications (e.g., rRNA operons), variable number tandem repeats or transposable elements that can prevent genome assembly. If these repetitive elements are longer than the library insert size a reliable assembly cannot be obtained (Magoc et al., 2013; Treangen & Salzberg, 2012). Thus, complete genome assembly should be possible using short read sequencing technologies, yet it is currently unknown what percentage of total phage isolates can be fully assembled, what the minimum coverage required for assembly is, and how likely they are to contain platform-specific assembly errors. Previous research has comprehensively evaluated the effect of both assembly program and depth of sequencing coverage for bacterial genomes (Magoc et al., 2013). However, the likelihood of a successful phage assembly, as well as the factors that may affect assembly, remain unknown. We aim to determine how the choice of assembler, depth of sequencing, and how multiplexing phages will influence the likelihood of a successful genome assembly.

## Materials and Methods

Phage T4 DNA was purchased from Fluka. Using a CsCl purified stock of phage HP1, DNA was extracted. Cyanophage S-PM2d and S-RSM4 were cultured as previously described (Clokie et al., 2003). The remaining phage isolates were cultured on their respective hosts in King’s B medium (King, Ward & Raney, 1954) at 28 °C, purified with CHCl_3_ and stored at 4 °C.

### DNA extraction

Phage DNA was extracted from 1 mL of fresh lysate by a modified phenol:chloroform method (Clokie et al., 2003). Briefly, cell debris was pelleted by centrifugation at 13,000× *g* for 10 min at 4 °C. The supernatant was extracted, transferred to a fresh tube, and the process repeated. The final supernatant was mixed with an equal volume of phenol (pH 10) and vortexed for 30 s prior to centrifugation at 13,000× *g* for 10 min at 4 °C. The aqueous layer was mixed with an equal volume of phenol:chloroform (1:1) and vortexed for 30 s prior to centrifugation at 13,000× *g* for 10 min at 4 °C. Finally, the aqueous layer was extracted, mixed with an equal volume of phenol:chloroform:isoamylalcohol (25:24:1), and vortexed for 30 s prior to centrifugation at 13,000× *g* for 10 min at 4 °C. The aqueous layer was extracted again, mixed with 1/10th volume 7.5 M ammonium acetate, and two volumes of ice cold 100% ethanol prior to centrifugation at 13,000× *g* for 30 min at 4 °C. DNA was precipitated at −20 °C. The DNA pellet was washed twice in 70% ethanol, dried, and resuspended in nuclease-free water prior to quantification with Qubit (Life Technologies). DNA was diluted to 0.2 ng μl^−1^ and libraries prepared using the NexteraXT (Illumina) protocol following the manufacturer’s instructions.

## Bioinformatics Analysis

Phage genomes were downloaded from EBI in February 2013 and filtered to remove any genomes that contained unknown or ambiguous bases, resulting in 1826 genomes that were used for creating in silico datasets (see Table S1 for accession numbers). Simulated datasets of 2 × 300 bp paired-end reads were produced using ART Illumina 2.1.8 (Huang et al., 2012). Read quality profiles for input into ART were generated from a previous MiSeq run and produced error model profiles that are included as supplementary data. Insertion and deletion rates for in silico reads were based on the same read quality profiles. For initial comparison of assembly programs, 300 bp read sets were produced at 100× coverage of each genome, with a mean insert size of 300 bp. For further analysis, read sets were produced for a coverage of 20, 30, 40, 50 and 100×. Insert sizes of 300, 500 and 650 bp were used with selected datasets. Each set of reads was assembled with SPAdes v3.1 (Bankevich et al., 2012), Velvet v1.2.10 (Zerbino & Birney, 2008), and Ray v2.3.1 (Boisvert, Laviolette & Corbeil, 2010). For SPAdes the ‘–only-assembler’ parameter was used. For Velvet, a range of kmer values were tested with VelvetOptimiser using the parameters ‘-s 51 –e 199 –× 20 –cov_cutoff 4’. For Ray, the parameter ‘-k 61’ was used. All other parameters in each assembler were left at default settings. Genome assembly was assessed using QUAST (Gurevich et al., 2013) against the reference genome used for production of the reads. Genomes were defined as complete if a single contig assembled without error and covered > 97% of the reference sequence, as determined by QUAST (Gurevich et al., 2013). Partial assemblies did not meet the required 97% threshold and had no assembly errors. Misassemblies were defined as genomes that contained errors, as identified by QUAST analysis. To simulate the pooled libraries, genomes were randomly sampled with replacement from specific sets of phage genomes, either using all phage genomes or only those confined to a particular host (e.g., *Pseudomonas*, *Mycobacterium*, *Synechococcus* and *Bacillus*) prior to de novo genome assembly.

### De novo assembly of phage isolates

Reads were trimmed with Sickle (Joshi & Fass, 2011) using default parameters. Genomes were assembled using SPAdes as described above. Reads were mapped back against the resulting contigs using BWA MEM v0.7.5 (Li, 2013) to check for assembly errors. Manipulation of SAM and BAM files was performed with SAMtools (Li et al., 2009). BAM files were processed with Qualimap v0.7.1 (García-Alcalde et al., 2012) to calculate the read coverage per contig and percentage of non-phage reads per sample. Reads for S-PM2d and T4 were submitted to the EBI archive under accession numbers PRJEB9935 and PRJEB9928 respectively. Reads and assemblies were submitted for HP1 [PRJEB9930], HC15b1 [PRJEB11092], HC15b2 [PRJEB11762], VCM1a [PRJEB11093], VCM1b [PRJEB11761], T17A [PRJEB11094], HC15g [PRJEB11095], HC4a [PRJEB11096], and AM-2105 [PRJEB11760]. The bacterial host of phages HC15g and HC4a has yet to be confirmed; putative hosts were identified at the genus level by analysing the contaminating host DNA within the phage DNA samples using Kraken (Wood & Salzberg, 2014).

### SNP and INDEL calling

Reads were mapped against a reference genome using BWA MEM (Li, 2013). Manipulation of SAM and BAM files was performed with SAMtools (Li et al., 2009). An mpileup file was produced using the −B option and the resulting file used with VarScan v2.3 (Koboldt et al., 2012) for both SNP and INDEL calling at a minimum average quality of 30, minimum variant frequency of 90%, with a minimum coverage of 30.

## Results

To ascertain if the use of Illumina sequencing technologies is a suitable method for the high-throughput assembly of phage genomes, an artificial dataset was constructed from all high quality genomes (1826) in the EBI database at the time of analysis (http://www.ebi.ac.uk/genomes/phage.html). Genomes were assembled using three commonly used assembly programs (Velvet, SPAdes, and Ray) at a sequencing coverage of 100× to determine if the algorithm affects the successful assembly of phage genomes compared to the original reference genome. QUAST was used to assess the assembly parameters compared to the original assembly. Genomes were assessed on their completeness (% of genome on a single contig) and the number of assembly errors (Table S1). This resulted in 1800, 1545 and 1638 complete error free genomes, using SPAdes, Velvet, and Ray respectively (Table 1). A greater proportion of genomes were assembled with SPAdes, compared to both Velvet and Ray (Table 2). Velvet produced an increased proportion of assemblies that were misassembled, compared to the reference.

**Table 1:** Phage-host systems used in this study.

Phage	Host	Reference
HC15b	*Pseudomonas syringae* pv. aesculi	This study
VCM1	*Pseudomonas syringae* pv. tomato DC3000	This study
SHL2	*Pseudomonas syringae* pv. tomato DC3000	This study
T17A	*Pseudomonas syringae* pv. tomato PT23	This study
HC15g	21.1.2 (*Pantoea* sp)	This study
HC4a	21.1.2 (*Clavibacter* sp)	This study
S-PM2	*Synechococcus* sp WH7803	(Puxty et al., 2015)
S-RSM4	*Synechococcus* sp WH7803	(Millard et al., 2009)
HP1	*Haemophilus influenzae*	(Esposito et al., 1996)
T4	*Escherichia coli*	(Miller et al., 2003)

**Table 2:** Assembly of 1826 phage genomes with three assembly algorithms.

	SPAdes	Velvet	Ray
Complete assembly	98.6% (1800)	84.6% (1545)	89.7% (1638)
Incomplete assembly (no errors)	1.4% (26)	11.4% (208)	9.3% (170)
Assembly with errors	0	4.0% (73)	1.0% (18)

SPAdes provided the highest successful genome assembly at 100× coverage with an insert size of 300 bp; therefore, further parameters were tested using this assembler. Different levels of coverage (20, 30, 40, 50 and 100×) were then tested for each phage.

The ability to assemble genomes without misassembly is essential, if genome structure and synteny between phages is to be studied. The use of in silico reads from genomes that have previously been assembled allows the likelihood of misassemblies to be assessed. No misassemblies were found at 20–100× coverage, confirming that it is possible to correctly assemble phage genomes using SPAdes.

**Figure 1:**
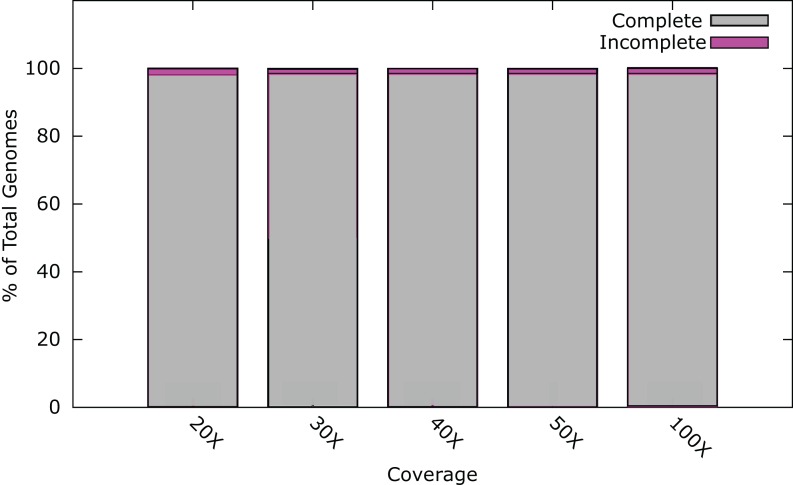
Percentage of phage genomes that were correctly assembled into a single contig at differing fold coverage of the genome. A total of 1826 phage genomes were assembled using SPAdes v3.1 (Bankevich et al., 2012) with an insert size of 300 bp at 100× coverage.

At 20× coverage, 98.0% of all phage genomes were assembled into a single contig (Fig. 1). At 30× coverage this increased to 98.5% (1800); increasing the coverage above 30×, provided no benefit to the assembly outcome (Fig. 1). Twenty-six phage genomes did not assemble completely even at 100× coverage, these phages had a range of genome sizes, %GC content, and different hosts (Table S2). Phages that did not assemble, had an average genome size of 121 kb, larger than phages that did assemble, with an average genome size of 66 kb. In order to try and successfully assemble these phage genomes into single contigs, we further investigated insert size and fold coverage in a range of combinations (Table S2). Insert size was increased to 650 bp and fold coverage to 1,000× (Table S2), resulting in the completion of one additional genome. The reason for the incomplete assembly in most instances is the failure to assemble the ends of the genome onto a single contig. For most genomes > 90% of the genome was assembled as a single contig, but this did not reach the 97% threshold (Table S2). Analysis of the genomes with ABACAS v1.03 (Assefa et al., 2009) against the known reference genome did not reveal any repeat regions that would prevent assembly.

These results indicate that it is possible to de novo assemble the genomes of the majority of phages correctly utilising current Illumina technology, under the condition that each phage sample was prepared as a separate sequence library. However, with a typical MiSeq run output being ~25 million paired-end reads, using a 96 multiplex strategy with dual indexes would result in an average coverage of > 1,000× of a 100 kb phage genome, or ~400× coverage using 384 indexes. A single MiSeq run has the capacity to sequence and assemble many times this number at 30× coverage. This capacity could be utilised through custom barcodes allowing the indexing of more samples. Such an approach carries with it the increased associated cost of library preparations using a larger number of indexes. An alternative approach is to pool phage genomes into a single library preparation. However, with de novo assembly, there is the possibility that the phages will be too similar to assemble into individual genomes post-sequencing.

In order to rule out the possibility of misassembly due to the similarity of different phages, two phage genomes were sampled (with replacement) from the 1820 phages previously assembled (Table S4), producing a model read set for each phage genome at an even coverage of 20×. This was repeated 960 times, thus simulating the output of 10 MiSeq runs. Combining two phages within a single library resulted in the complete assembly of both phage genomes in 98.8% of the samples (Table 3), when an even coverage was used with each phage genome. Misassembly occurred for eight genomes, when the phages that were combined were from the same or closely related bacterial hosts (Table 4).

**Table 3:** Assembly of 1920 phage genomes from 960 pairs of genomes combined at random in silico. An insert size of 300 bp and 20× coverage was used.

Completely assembled genomes (%)	Incomplete genome assembly (%)	Misassembled genomes (%)
98.8 (1897/1920)	0.78 (15/1920)	0.42 (8/1920)

**Table 4:** Genome properties of phage combinations that did not allow complete genome assembly.

Phage 1			Phage 2		
Accession	Genome size (kb)	Host	Accession	Genome Size (kb)	Host
JN020140	50.988	*Mycobacterium*	KJ174156	52.136	*Mycobacterium*
AY954960	43.576	*Staphylococcus*	JX013863	45.242	*Staphylococcus*
AF323669	41.834	*Lactococcus*	DQ394808	35.992	*Lactococcus*
KF562100	76.323	*Mycobacterium*	JF937101	109.086	*Mycobacterium*

To further test how co-sequencing genomes of similar phages will impact their assembly, additional simulations were carried out using combinations of sequences from phages isolated from the same bacterial host. Due to their numerical abundance in the current EBI dataset, phages infecting *Mycobacterium* (387 genomes) and *Pseudomonas* (142 genomes) were chosen for these simulations. Working on a basis of 96 libraries per MiSeq run, 96 pairs of *Pseudomonas* and *Mycobacterium* genomes were randomly sampled and combined, model reads were produced and genomes were assembled. Reads were combined at different sequencing ratios, with a minimum of 30× coverage at various ratios (1:1, 9:1, 1:9, and two randomly selected coverages). For both *Mycobacterium* and *Pseudomonas* phages, the effect of using different ratios of sequencing depth (compared to even coverage for both samples) was minimal; for *Mycobacterium* phages one extra genome was assembled at a 1:1 ratio compared to ratios of 9:1 or 1:9, while for *Pseudomonas* phages there was no difference in assembly outcome (Tables S5 and S6). For *Mycobacterium* phages it was possible to correctly assemble 166/192 genomes and partially assemble five genomes, with 21 genomes containing assembly errors (Table 5). For *Pseudomonas* phages, 171/192 phage genomes were correctly assembled, eight partially assembled and 13 contained assembly errors (Table 5). When compared to the random sampling of all phages, there was a clear increase in the proportion of phages that contained assembly errors when phages from the same host were combined. This finding presents a substantial issue when multiplexing novel phage genomes from the same host, since these assembly errors are more difficult to identify without prior knowledge of the genome sequence.

**Table 5:** In silico assembly of phage genomes when 192 phage genomes were assembled from 96 libraries each containing two genomes.

	Fully assembled genomes, no misassembly (%)	Partially assembled genomes, no misassembly (%)	Genomes with assembly errors (%)
*Mycobacterium* phages	86.5 (166/192)	2.6 (5/192)	10.9 (21/192)
*Pseudomonas* phages	89.1 (171/192)	4.2 (8/192)	6.8 (13/192)
*Mycobacterium* & *Pseudomonas* phages	100 (192/192)	0 (0/192)	0 (0/192)
*Mycobacterium* & *Pseudomonas* & *Synechococcus* phages	93.4 (269/288)	6.6 (19/288)	0 (0/288)
*Mycobacterium* & *Pseudomonas* & *Synechococcus & Bacillus* phages	91.4 (351/384)	8.6 (33/384)	0 (0/384)

An alternative strategy for the high-throughput sequencing of multiple phages would be to isolate them from more than one host and combine them in a single library for sequencing. This was tested in silico, using *Mycobacterium* and *Pseudomonas* phages. This approach allowed the complete assembly of all 196 phage genomes without any assembly errors (Table 4). In order to test the limits of this multiplexing approach, this basic principle was extended in silico by production of read sets for mixtures of phages from three hosts (*Mycobacterium*, *Pseudomonas* and *Synechococcus,* see Table S7) and four hosts (*Mycobacterium*, *Pseudomonas*, *Synechococcus* and *Bacillus,* see Table S8). When combining three or four phages in a single sample, the proportion of phage genomes that were partially assembled increased with the complexity of the mixture, but crucially the number of misassemblies did not (Table 4).

**Figure 2:**
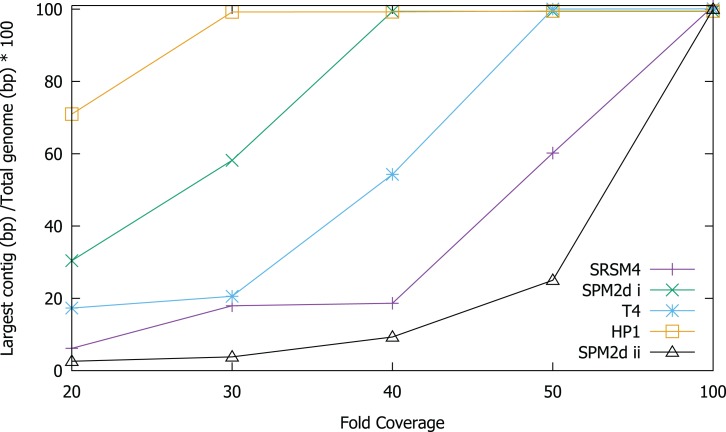
Assembly of T4, S-RSM4, S-PM2d and HP1 phage genomes at different sequencing depths. Assembly was assessed as the size of the largest contig, calculated as a percentage of reference genome size. SPM2di and SPM2dii represent different library preparations of cyanophage S-PM2d.

**Figure 3:**
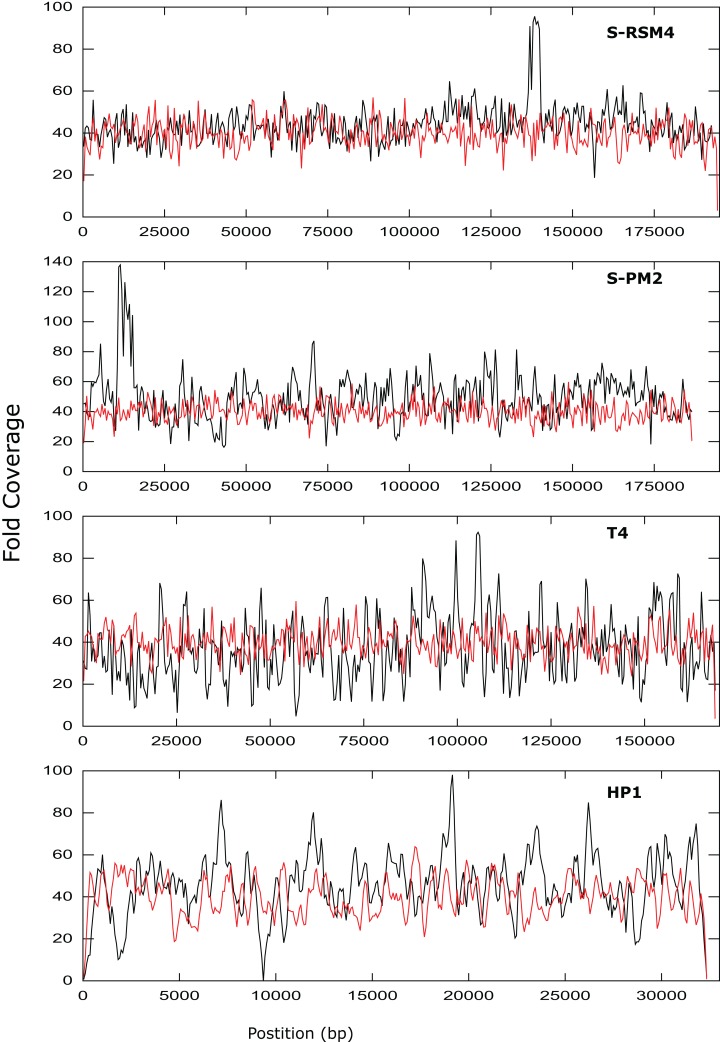
Coverage across the genomes of S-PM2, S-RSM4, T4 and HP1. Reads were mapped against the reference genomes of S-PM2d (accession: LN828717), S-RSM4 (accession: NC_013085.1), T4 (accession: AF_158101) and HP1 (accession: NC_001697.1). The data is representative of an average 40× coverage. Red lines are in silico reads, black lines are sequence reads.

In order to test the validity of the predictions reached via in silico experiments, we re-sequenced four known phages and compared the results of sequencing to the in silico predictions. We determined the ability to assemble each genome at a range of different sequencing depths by sub-sampling (Fig. 2). Phage T4 genome assembly required a threshold of 50× coverage and the median insert size was 350 bp (Fig. 3). In silico reads with an insert size of 300 bp allowed complete assembly of phage T4 at 20× coverage. Read mapping to the published T4 genome (accession AF158101) revealed 156 SNPs and 55 short INDELs (Tables S10 and S11). The large number of apparent mutations was initially surprising. However, the sequenced T4 DNA was commercially produced (Fluka) and the provenance of the T4 used in its production is unknown. The choice of reference will clearly affect the SNPs that are detected. There are currently seven complete phage T4 genomes (sensu lato); RB55 (KM607002), RB59 (KM607003), T4 (AF158101), T4 strain 147 (KJ477685), T4 strain GT7 (KJ477686), T4 strain wild (KJ477684) and T4T (HM137666), all of which have slightly different genome sizes. Each could serve as reference for SNP calling and produce differing results. For instance, T4 genes *g10*, *g12* and *g13* (Table S10) were all found to have at least one non-synonymous mutation, however, these would not have been called SNPs if T4T (HM137666) was used as reference. The identification of so many SNPs highlights the micro-diversity of what is referred to as phage sensu lato T4.

It was possible to assemble phage HP1 at 30× coverage, although coverage at the termini of the genome was strikingly lower, compared to the rest of the genome (Fig. 3). Additionally, a region with no coverage can be clearly identified at ~9,000 bp. Further investigation of the mapped reads identified a deleted region located at 9,287–9,406 bp when compared to the reference strain (accession: U24159). This causes an in-frame deletion of 39 amino acids from the sequence of the HP1p17 protein; whether the resulting protein is still functional remains unknown. Forty-three SNPs and six INDELs were identified in addition to the large deletion (Table S13). Twenty-nine SNPs caused non-synonymous mutations, eight were synonymous mutations and six were in intergenic regions (Table S12). Of the six INDELs that were identified, three occur in HP1p22, encoding a capsid scaffolding protein (Esposito et al., 1996) (Table S12). Although scaffolding proteins do not appear in the final capsid, they play an important role in its correct assembly. HP1p22 is a homologue of the GpO protein of P2, which is essential for the production of proheads (Lengyel et al., 1973). Therefore, despite the numerous INDELs and SNPs, this essential structural protein appears to still be functional, since this genome produced functional phages.

Cyanophage S-PM2d was assembled from two independent libraries from different DNA extractions, with a median insert size of 362 bp and 139 bp, producing assemblies designated S-PM2di and S-PM2dii respectively (Table 4). The larger insert library allowed assembly into a single contig at 50× coverage (Fig. 3), which was higher than the 20× coverage predicted by our in silico experiments. The 139 bp insert library required 100× fold coverage for complete genome assembly.

Whilst there was similar coverage at the termini compared to the rest of the genome, there was approximately double the number of reads that were mapped to the region 10,500–12,000 bp (Fig. 3). A similar pattern was observed for cyanophage S-RSM4, where a higher proportion of reads mapped to the region 1,37,800–1,39,000 bp. Assembly of cyanophage S-RSM4 was possible at 100× coverage (Fig. 2). Despite having similar %GC content and genome sizes (Table 6), the insert size of libraries for S-PM2i, S-PM2ii and S-RSM4 were very different, with median insert sizes of 362, 139 and 155 bp respectively (Table 6). Both S-PM2d and S-RMS4 contained no SNPs or small INDELs when compared to the published reference strains (accession LN828717 and FM207411).

**Table 6:** Assembly statistics for phage isolates from de novo assembly.

Phage	% Phage reads	% Non-phage reads	Insert size (mean/median) [bp]	% GC	Genome size [bp]
T418	99.3	0.7	380 349	35.3	168,903
S-RSM4	76.84	23.17	196 155	41.1	194,454
S-PM2dii	78.8	22.2	218 139	37.8	186,736
S-PM2di	52.31	47.69	379 362	37.8	186,736
HP1	99.99	0.01	374 325	40.0	32,355
HC15b1	1.72	98.21^*^	390 365	49.6	48,452
HC15b2	96.40	3.60	397 373	56.6	40,346
SHL2	93.47	6.53	233 153	56.6	40,466
HC15g	99.03	0.07	420 399	49.6	48,452
HC4a	93.39	6.91	485 455	65.1	48,214
VCM1a	2.35	97.65^&^	343 305	48.5	98,765
VCM1b	90.29	9.71	339 306	57.3	40,402
T17a	98.94	1.06	446 417	58.0	40,242
AM-2015	0.49	99.51^$^	218 336	55.07	96,840

**Notes:**

* inclusive of reads that map to HC15b2.

& inclusive of reads that map to VCM1b.

$ inclusive of reads that map to S-PM2d.

The re-sequencing of four phages clearly demonstrates that complete de novo assembly of phage genomes from Illumina sequencing data is possible at coverage levels readily achievable on the MiSeq. For high-throughput phage genome sequencing, it will be necessary to remove other bottlenecks in the process. Standard NexteraXT library preparation only requires one ng of DNA for library preparation, as the protocol utilises transposons carrying adapter oligos to simultaneously fragment and ligate adapters in a single reaction (Marine et al., 2011). Researchers have optimised phage purification methods that use a column-based approach rather than time-consuming CsCl gradients (Henn et al., 2010). In this study, an alternative approach eliminated expensive columns and required only minimal phage lysate. This was achieved using a basic phenol:chloroform extraction (see methods) on a crude phage lysate.

At least one phage genome was assembled from each lysate (Table 6), with two genomes arising from the lysates of S-PM2dii, HC15b and VCM1. Assemblies used a minimum of ~1,40,000 reads, well in excess of the 30× coverage predicted using the in silico reads. After assembly, the total read pool was sub-sampled at random to produce datasets with coverage ranging from 20–100×. 30× coverage was sufficient to assemble complete genomes for seven of the nine novel phages, and the remaining two genomes assembled at 40 and 50× respectively (Fig. S1). In addition, 16 phage datasets produced using Illumina technology were extracted from the ENA (www.ebi.ac.uk/ena) and assembled at 20, 30, 40, 50 and 100× coverage (Fig. S2). All 16 of these genomes assembled as a single contig at 30× coverage.

Contaminating non-phage reads were identified by their failure to map to assembled phage genomes. The proportion of reads that could not be mapped to the phage genome varied from 0.01–47.69% (Table 6). In the case of S-PM2ii, somewhat surprisingly, two phage genomes were assembled from the lysate. The first was S-PM2d (~187 kb) and the second a genome of ~96.84 kb. The latter phage is of unknown origin, and not a cyanophage based on its lack of genetic similarity to known cyanophages. Furthermore, phylogenetic analysis of *phoH*, a common phage marker gene (Goldsmith et al., 2011), indicated that the closest *phoH*-containing relative is a phage infecting *Caulobacter*, not a cyanobacterium (Fig. S3). This phage is probably a result of a low-level contaminant in the stock of S-PM2d that was only detected due to the very high depth of initial sequencing coverage (> 2,000×). This contaminant has not been detected previously in S-PM2d which was recently purified by three rounds of plaque assay and sequenced to a high depth of coverage (Puxty et al., 2015).

## Discussion

In silico predictions suggest that it is possible to correctly assemble the vast majority of phage genomes at 100× when using three common assembly programs. Using default setting with SPAdes, a greater number of genomes was assembled compared to using either Velvet or Ray. It is possible that further optimisation of parameters for both Ray and Velvet would increase the number of complete genomes. Further testing of coverage and insert size was carried out using SPAdes, but the results are likely to prove useful as a starting point for other assembly programs. Utilising SPAdes we found that increasing coverage above 30× resulted in a small increase of 0.5% in assembly success. However, 26 genomes were found not to assemble, even when a coverage of 1,000× or a large insert of 650 bp was used. Unlike their bacterial hosts, phages do not contain large repeats and multiple gene duplications that make bacteria recalcitrant to complete assembly (Ricker, Qian & Fulthorpe, 2012). For those genomes that were not assembled, it was the ends of the genome (as defined by the reference genome) that remained incomplete.

Whilst the use of NexteraXT has many benefits, the ability to consistently produce an insert size of > 500 bp for genomes of unknown size and %GC content is likely an unattainable target. As seen by the S-PM2d libraries tested in this study, the insert size was inconsistent, despite following the same protocol. Instead of trying to control the insert size, adjusting the genome coverage is a more attainable target, and one which can overcome very short insert sizes. Our experimental data showed that a higher coverage than predicted in silico was required to achieve complete genome assembly for some genomes. There are several possible explanations for this finding: (1) The reads that are produced in silico are completely random, unlike experimental data where some regions of the genome are clearly over-represented (Fig. 3); (2) The mean insert sizes of these experimental libraries was smaller than our in silico libraries (for example, in S-PM2, when the coverage required for assembly decreased from 100–40×; (Fig. 2); (3) A high proportion of non-phage DNA results in lower fold coverage than predicted, as was the case for S-PM2d and S-RSM4.

Using experimental read data from 13 phages in this study and 16 genomes retrieved from the ENA, we found that 82.75% (24/29) of these genomes could be assembled at 30× coverage, compared to the in silico data of 98.5% of genomes. Increasing phage sequencing throughput, by combining multiple phages within a single library preparation, would allow doubling of sequencing capacity at a marginal increase in cost for DNA extraction. The results of in silico predictions suggest that this would be a useful strategy if the phages were isolated from multiple hosts. This approach has previously been successfully used to assess the diversity of phage from the North Sea (Wichels et al., 1998). The recent development of a method of single-plaque phage sequencing (Kot et al., 2014), optimisations of the NexteraXT protocol to reduce the cost per library (Baym et al., 2015), and the results of this work all indicate it should be possible to take a high throughput approach to phage genome sequencing.

S-PM2d resequencing has confirmed, by chance, that it is possible to completely assemble the genomes of two phages from different hosts. Detection of the novel phage AM-2015 while sequencing S-PM2, was possible due to the extremely high sequencing depth used in this study, as it accounted for only ~13% of reads in the initial library. Similarly, in the case of phages VCM1 and HC15b, sequencing at high depth has revealed the presence of an additional phage in each of the lysates. All of these phages had undergone plaque purification prior to production of lysates. The presence of multiple phages within plaque purified isolates has previously been reported (Henn et al., 2010) and are known to be from the spontaneous release of a host prophage (Cowley et al., 2015). Without having complete host genomes for strains used in this study, it is not possible to determine if the presence of multiple phages is due to prophage release from the host.

Whilst it was possible to assemble multiple phage genomes from a single library in silico, simultaneous sequencing of multiple phages that are closely related could result in misassemblies that are extremely difficult to detect. Therefore, complete phage genomes assembled from metagenomes should be interpreted with care. We used SPAdes for genome assembly in this study, but acknowledge that it is not recommended for metagenome assembly. However, it seems likely that the misassembly of closely related phage genomes would result independent of the choice of assembler. When combining multiple phages from different hosts there was no increase in the number of misassembles, with a small increase in the percentage of incomplete genomes. Therefore, the multiplexing of phages from different hosts in a single library represents an efficient and cost effective way to increase the throughput of phage genome sequencing.

### Genome discussion

Phages T4, S-PM2d and S-RSM4 are myoviruses with a circularly permuted genome that are packaged in a headful mechanism. In the case of T4, 103% of the genome is packaged (Alam et al., 2008). While mapping reads back against T4, S-PM2 and S-RSM4, we observed that coverage did not decrease dramatically at the genome’s ends. This is entirely consistent with what would be expected from circularly permuted genomes, whereby a population of phage particles will all package > 100% of their genome and have different terminal ends. For S-PM2d and S-RSM4, a discrete region of the genome was identified that was significantly over-represented compared to the rest of the genome (Fig. 3). This has been observed previously for the cyanophage S-PM2, whereby a large deletion adjacent to this region has occurred (Puxty et al., 2015). Its presence in S-RSM4 suggests it may be a feature common to cyanophages. Whilst increased coverage at specific regions was observed for both cyanophages, the over-represented region of S-PM2 has no sequence similarity to any region in S-RSM4 and does not contain repeated units. What causes these regions to occur is unknown; the lack of any regions that have zero coverage indicates these phages do not have exact terminal repeats or cohesive termini as continuous coverage across the genome can only be observed for circularly permuted genomes using NexteraXT library preparations. Yet, the high coverage clearly demonstrates the enrichment of specific regions within the population of virions sequenced. Whether this enrichment is a sequence feature that causes them to be packaged more frequently or duplications of the genes in this region giving virions a fitness advantage, thus causing them to become dominant within a population, remains unknown.

In contrast to the above *T4likeviruses*, HP1 is not circularly permuted and instead has cohesive termini (Esposito et al., 1996; Fitzmaurice et al., 1984). The coverage map of the HP1 genome clearly shows a decrease in the sequencing coverage of terminal regions, a feature absent in T4, S-PM2 and S-RSM4 (Fig. 3). The distinct ends of the cohesive termini are less likely to have transposons inserted within them, whereby the population of different termini in a circularly permuted genome will give rise to a more evenly distributed coverage of reads at the termini (as was observed). Decreased coverage at terminal regions seems to be a unique property of non-circularly permuted genomes and can be used as a marker for their distinction from phages with circularly permuted genomes. The use of NexteraXT transposon based library preparation does mean that the genomes that are linear will never have the exact termini sequenced, as it is impossible for transposon to insert upstream of a terminal base. This can be avoided by the use of library preparation kits that are not transposon based, but at the cost of more hands-on time for library preparation. The choice will depend on the research question; terminal sequences may not be necessary for studies of gene content, for example.

## Conclusions

This work has demonstrated it is possible to assemble the vast majority of phage genomes using short read sequencing technologies in silico. Using 30× coverage was sufficient to assemble the majority of phage genomes both in silico and from experimental data. However, due to the uneven coverage produced by either the NexteraXT preparation or biological properties of the phage genome, a coverage of 100× would be recommended as a starting point to maximise the likelihood of successful assembly in a high-throughput manner. In silico analysis predicts that pooling two, three, or even four phages from different hosts into a single NexteraXT library preparation can increase the throughput of phage genome sequencing, without increasing misassemblies or additional costs for increased barcodes and library preparations. The current bottleneck in phage genomics is now clearly the ability to culture phages and isolate their DNA, rather than the capacity to sequence or assemble them.

## Supplemental Information

10.7717/peerj.2055/supp-1 Supplemental Information 1 Supplementary Tables S1–S14. Table S1. Assembly of 1826 bacteriophage at 100× coverage, with the three assembly programs SPAdes3-1, Velvet and Ray. Table S2. Results of assembling 26 bacteriophage with SPAdes3-1 using an increasing insert size from 300–650 bp and sequencing depth in the range of 20–1,000×. Table S3. Assembly data of 1826 bacteriophages assembled with SPAdes3-1 at 20, 30, 40, 50 and 100× coverage. Table S4. Assembly data of 960 pairs of bacteriophage assembled with SPAdes3-1. Table S5. Assembly data of 96 pairs of *Pseudomonas* bacteriophages assembled with SPAdes3-1. A minimum of 30× coverage was maintained for all samples. Differing ratios of coverage were tested for each combination of phages, with ratios of 1:9, 1:1, 9:1 and two randomly selected coverages. Table S6. Assembly data of 96 pairs of *Mycobacterium* phages assembled with SPAdes3-1. A minimum of 30× coverage was maintained for all samples. Differing ratios of coverage were tested for each combination of phages, with ratios of 1:9, 1:1, 9:1 and two randomly selected coverages. Table S7. Assembly data of 96 combinations of *Pseudomonas and Mycobacterium* phages assembled with SPAdes3-1. A minimum of 30× coverage was maintained for all samples. Differing ratios of coverage were tested for each combination of phages, with ratios of 1:9, 1:1, 9:1 and two randomly selected coverages. Table S8. SPAdes3-1 assembly data of 96 combinations of three bacteriophages infecting *Mycobacterium*, *Pseudomonas* and *Synechococcus*. A random coverage was selected for all samples with a minimum of 30× and maximum of 100×. Table S9. SPAdes3-1 assembly data of 96 combinations of four bacteriophages infecting infecting *Mycobacterium*, *Pseudomonas, Bacillus* and *Synechococcus*. A random coverage was selected for all samples with a minimum of 30× and maximum of 100×. Table S10. Identification of small indels by the re-sequencing of bacteriophage T4. Table S11. Identification of SNPs by the re-sequencing of bacteriophage T4. Table S12. Identification of SNPs by the re-sequencing of bacteriophage HP1. Table S13. Identification of small indels by the re-sequencing of bacteriophage HP1. Table S14. Phages extracted from the short read archives used to produce Fig. S1.

10.7717/peerj.2055/supp-2 Supplemental Information 2 Assembly data for nine bacteriophage isolates. Assembly of T17A, SHL2, HC15g, VCM1a, VCM1b, HC15-1, HC15-2, AM-2015 and HC4A bacteriophage genomes at different sequencing depths. [b]Assembly was assessed as the size of largest contig as a percentage of reference genome size.

10.7717/peerj.2055/supp-3 Supplemental Information 3 Assembly of 16 bacteriophage isolates extracted from the short read archive. Assembly of 16 bacteriophage isolates extracted from the short read archive.

10.7717/peerj.2055/supp-4 Supplemental Information 4 Phylogenetic analysis of phoH from bacteriophage AM-2015. Phylogentic analysis of phoH by Maximum Likelihood method, using the Le_Gascuel_2008 model of evolution. Bootstraps and branch lengths are based on 100 replicates. The analysis involved 101 amino acid sequences. All positions containing gaps and missing data were eliminated. There were a total of 187 positions in the final dataset.

10.7717/peerj.2055/supp-5 Supplemental Information 5 Supplementary Methods for phylogenetic methods. Supplementary Methods for phylogenetic methods.

10.7717/peerj.2055/supp-6 Supplemental Information 6 Error profile for read 2. Error profile for read 2, used in the generation of reads with art illumina.

10.7717/peerj.2055/supp-7 Supplemental Information 7 Error profile for read 1. Error profile for read 1, used for the generation of reads with art illumina.

## Additional Information and Declarations

### DNA Deposition

The following information was supplied regarding the deposition of DNA sequences: Genome accession numbers are located in Table S14 (deposited at http://www.ncbi.nlm.nih.gov/sra/).

### Data Deposition

The following information was supplied regarding data availability: The raw data has been supplied as Supplemental Dataset Files.
