# Supplementary figures and images for: Assessing Illumina technology for the high-throughput sequencing of bacteriophage genomes
Source: PeerJ. 2016 Jun 1;4:e2055. doi: 10.7717/peerj.2055 (PMC4893331; doi:10.7717/peerj.2055)

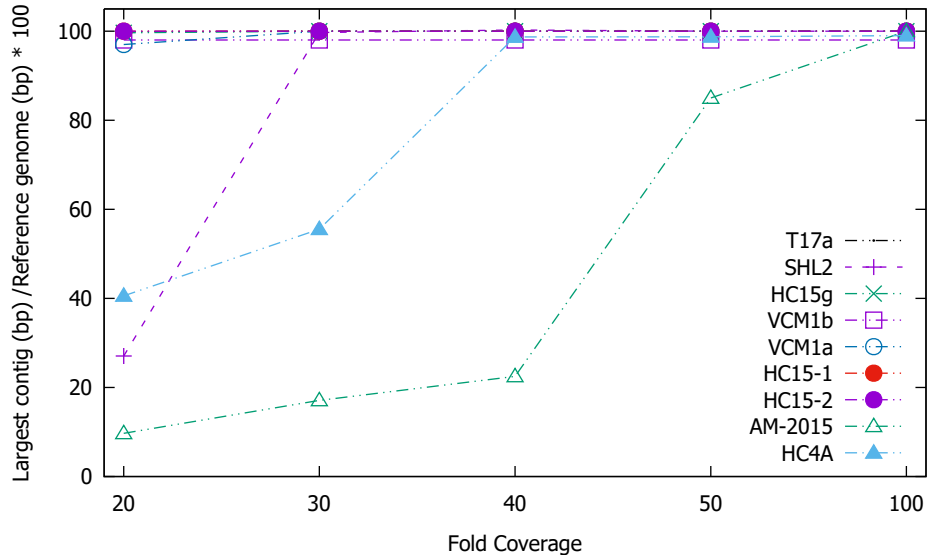

Supplement: Supplemental Information 2 — Assembly of T17A, SHL2, HC15g, VCM1a, VCM1b, HC15-1, HC15-2, AM-2015 and HC4A bacteriophage genomes at different sequencing depths. [b]Assembly was assessed as the size of largest contig as a percentage of reference genome size. [file peerj-04-2055-s002.pdf]

% of genome on a single contig

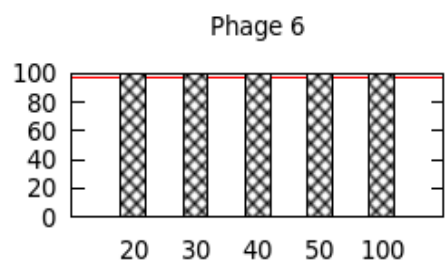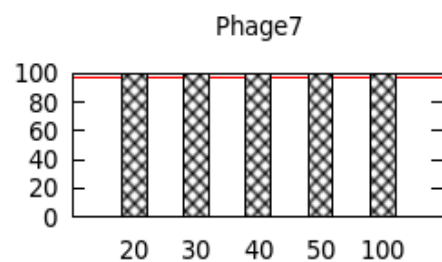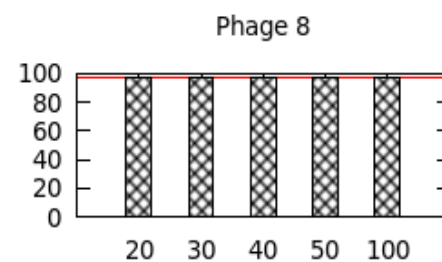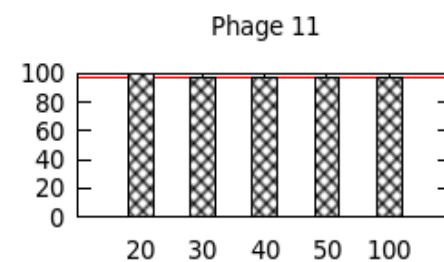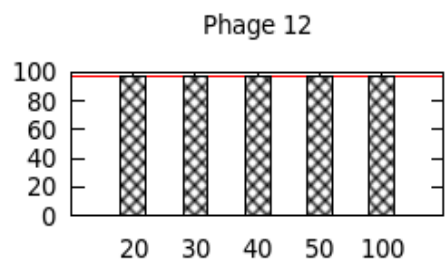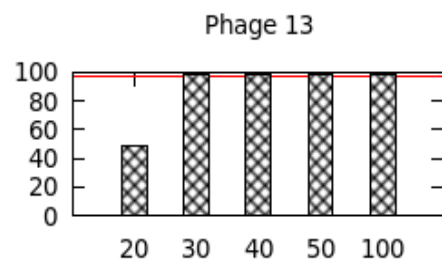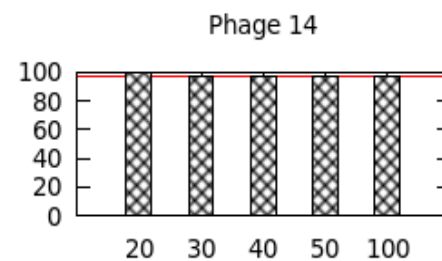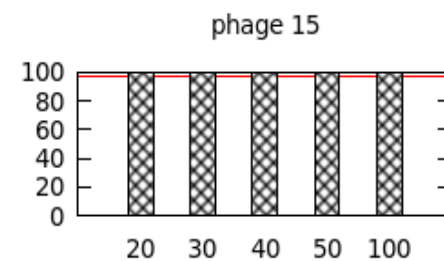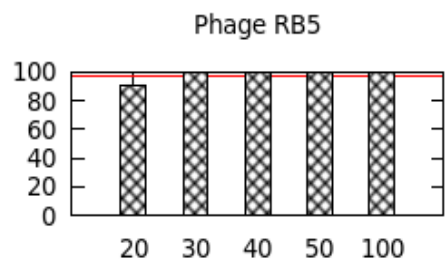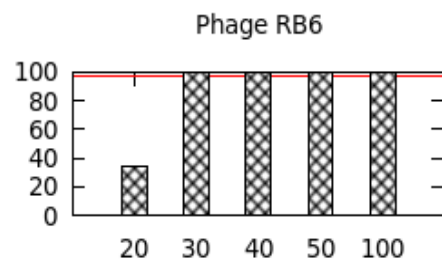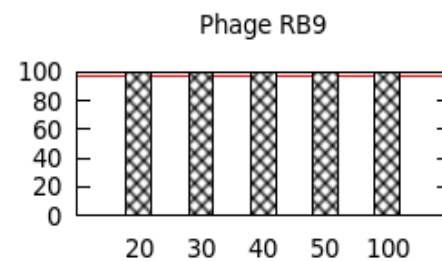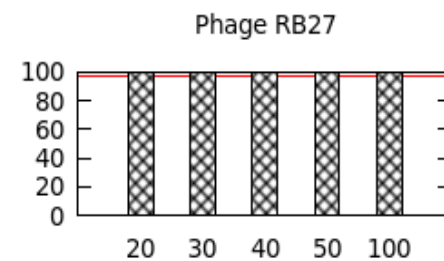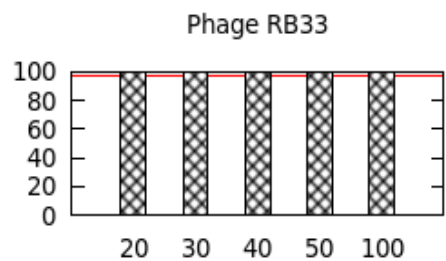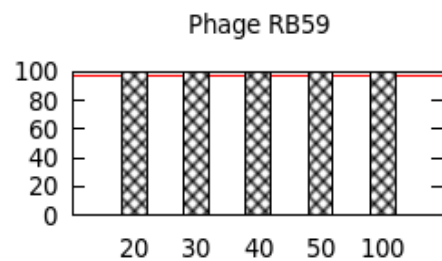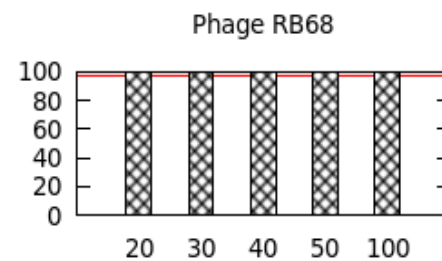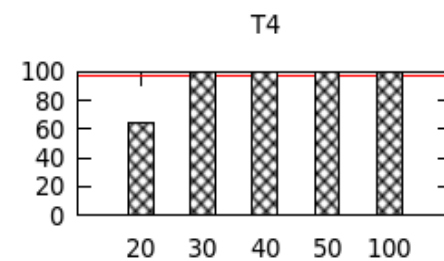

Sequencing depth

Supplement: Supplemental Information 3 — Assembly of 16 bacteriophage isolates extracted from the short read archive. [file peerj-04-2055-s003.pdf]

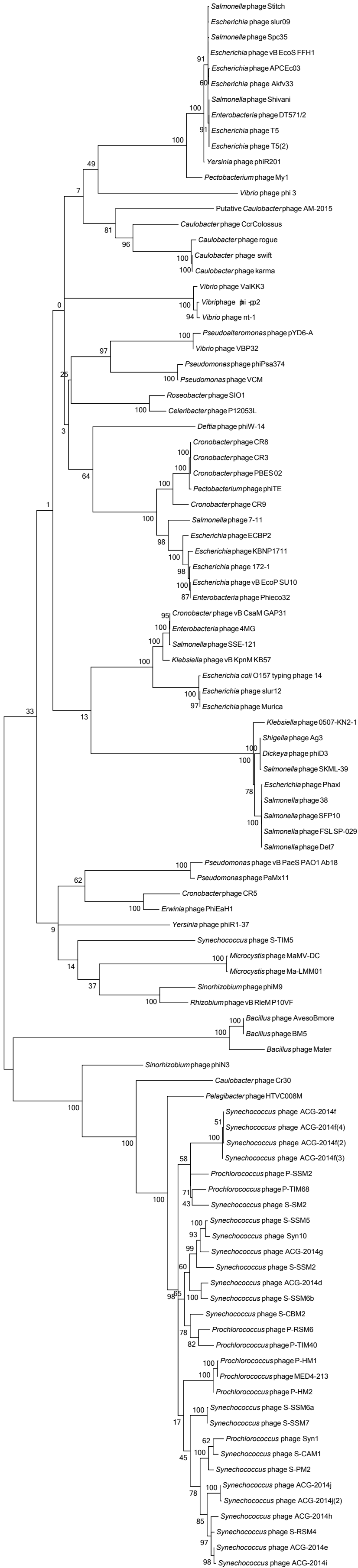

0.5

Supplement: Supplemental Information 4 — Phylogentic analysis of phoH by Maximum Likelihood method, using the Le_Gascuel_2008 model of evolution. Bootstraps and branch lengths are based on 100 replicates. The analysis involved 101 amino acid sequences. All positions containing gaps and missing data were eliminated. There were a total of 187 positions in the final dataset. [file peerj-04-2055-s004.pdf]
