# Supplementary material for: Assessing Illumina technology for the high-throughput sequencing of bacteriophage genomes
Source: PeerJ. 2016 Jun 1;4:e2055. doi: 10.7717/peerj.2055 (PMC4893331; doi:10.7717/peerj.2055)
Supplement: Supplemental Information 5 — Supplementary Methods for phylogenetic methods. [file peerj-04-2055-s005.docx]

Supplementary Methods

Phylogenetic analysis of phoH was carried out using MEGA (Kumar et al. 2008). Sequences were extracted from the nr database using BLAST, prior to alignment with MUSCLE (Edgar 2004) and trimming of the alignment with trimAI (Capella-Gutierrez et al. 2009). The most suitable model of evolution was selected with jmodeltest (Santorum et al. 2014). Further analysis was carried out in MEGA. 100 bootstraps were carried out to determine branch lengths and support values.

References

Capella-Gutierrez S, Silla-Martinez JM, and Gabaldon T. 2009. trimAl: a tool for automated alignment trimming in large-scale phylogenetic analyses. *Bioinformatics* 25:1972-1973. 10.1093/bioinformatics/btp348

Edgar RC. 2004. MUSCLE: multiple sequence alignment with high accuracy and high throughput. *Nucleic Acids Res* 32:1792-1797. 10.1093/nar/gkh340

Kumar S, Nei M, Dudley J, and Tamura K. 2008. MEGA: a biologist-centric software for evolutionary analysis of DNA and protein sequences. *Brief Bioinform* 9:299-306. 10.1093/bib/bbn017

Santorum JM, Darriba D, Taboada GL, and Posada D. 2014. jmodeltest.org: selection of nucleotide substitution models on the cloud. *Bioinformatics* 30:1310-1311. 10.1093/bioinformatics/btu032
